# Pretreatment Ki67-to-ADC Ratio Predicts Prognosis in Breast Cancer Patients Receiving Neoadjuvant Chemotherapy: A Retrospective Cohort Study

**DOI:** 10.3390/curroncol33090534

**Published:** 2026-09-02

**Authors:** Jun Fan, Lin Lin, Yang Tao, Yanjia Fan, Yudi Jin, Fajin Lv

**Affiliations:** 1 Department of Radiology, The First Affiliated Hospital of Chongqing Medical University, Chongqing 400016, China; fanjuncqmu@163.com (J.F.); linlincqmu@163.com (L.L.);; 2 Department of Breast and Thyroid Surgery, The First Affiliated Hospital of Chongqing Medical University, Chongqing 400016, China

**Keywords:** breast cancer, neoadjuvant chemotherapy, Ki67, ADC, δADC, prognosis, predictive model

## Abstract

(1) Background: Neoadjuvant chemotherapy (NAC) is important for breast cancer, but prognosis varies widely. Ki67 and apparent diffusion coefficient (ADC) reflect proliferation and cellularity, respectively. This study evaluated the prognostic value of the Ki67/ADC ratio (KA) and post-treatment ADC change (δADC) in breast cancer patients receiving NAC, and developed a survival prediction model incorporating these indicators. (2) Methods: Two cohorts of breast cancer patients treated with NAC were collected. Pre- and post-treatment breast MRI with diffusion-weighted imaging were obtained; ADC values were measured by two blinded radiologists. KA was calculated as pre-treatment Ki67 divided by pre-treatment ADC, and δADC as post-ADC minus pre-ADC. Disease-free survival (DFS) was the primary outcome. Cox regression and a predictive Cox model were used. (3) Results: A total of 419 patients were analyzed. Both KA and δADC were associated with survival. In multivariable analysis, KA remained an independent prognostic factor (HR 0.40, 95% CI 0.19–0.84, *p* = 0.015). High KA was associated with worse prognosis, particularly in patients without pathological complete response. The model incorporating KA showed better predictive performance than clinicopathological variables alone and effectively stratified high- vs. low-risk patients. (4) Conclusion: KA is a promising complementary biomarker for prognosis in breast cancer patients undergoing NAC. Its integration into a prognostic model improved survival risk prediction and may aid individualized post-treatment management.

## Simple Summary

For breast cancer patients receiving chemotherapy before surgery, survival outcomes differ greatly. We examined whether a combination of two simple measures, Ki67 and ADC, could help predict prognosis. In a study of 419 patients, we found that a higher ratio of Ki67 to ADC before treatment was linked to worse survival. Adding this ratio to a prediction model better identified high-risk patients than using usual clinical factors alone. This approach may assist doctors in personalizing post-treatment monitoring and decision-making for individual patients.

## 1. Introduction

Breast cancer remains one of the most common malignancies among women worldwide and is a leading cause of cancer-related mortality [1,2]. For patients with locally advanced or biologically aggressive disease, neoadjuvant chemotherapy (NAC) has become an important treatment strategy as it can reduce tumor burden, increase the rate of breast-conserving surgery, and provide an early assessment of treatment response [3,4]. Pathological complete response (pCR) is considered a favorable prognostic indicator, especially in aggressive subtypes such as HER2-positive and triple-negative breast cancer [5]. However, responses to NAC are highly heterogeneous, making the identification of robust biomarkers for long-term prognosis a top clinical priority.

Numerous studies have demonstrated that Ki67 expression correlates strongly with breast cancer tumor grade and predicts sensitivity to systemic therapy. Higher Ki67 expression often reflects faster tumor growth and may correlate with chemosensitivity in certain contexts [6]. Additionally, magnetic resonance imaging (MRI), particularly diffusion-weighted imaging (DWI), provides noninvasive information about tumor cellularity and microstructural changes [7,8]. The apparent diffusion coefficient (ADC) quantifies water molecule mobility within tissues and is inversely related to tumor cell density. Lower ADC values are often observed in more cellular and biologically active tumors [9].

Although Ki67 and ADC both reflect tumor biology from different perspectives, their combined value has not been fully explored. We hypothesized that the ratio of Ki67 to ADC before NAC, termed KA, may integrate proliferative activity and imaging-based cellular density, thereby providing a more comprehensive biomarker for prognosis. In addition, the change in ADC after treatment, δADC, may reflect treatment-induced microstructural alterations and be associated with pCR and survival.

In this study, we aimed to (1) evaluate the relationships between KA, δADC, and other clinicopathological characteristics in breast cancer patients receiving NAC; (2) explore the associations of KA and δADC with prognosis; and (3) construct and validate a predictive model based on KA or δADC to predict survival risk.

## 2. Materials and Methods

### 2.1. Patient Inclusion and Study Design

This retrospective study included the following two cohorts: (1) Cohort I, breast cancer patients who received NAC at the First Affiliated Hospital of Chongqing Medical University between January 2019 and January 2023; (2) Cohort II, breast cancer patients who received NAC at the First Affiliated Hospital of Chongqing Medical University between February 2023 and December 2023. The study protocol was approved by the institutional ethics committee (ID: 2025-286-01), and the requirement for informed consent was waived due to the retrospective nature of the analysis.

Inclusion criteria were as follows: (1) histologically confirmed invasive breast cancer; (2) received standard NAC followed by surgery; (3) available pre-treatment breast MRI, including DWI/ADC mapping; (4) available post-treatment MRI before surgery; (5) available Ki67 assessment from core needle biopsy material; and (6) complete clinicopathological and follow-up data.

Exclusion criteria included: (1) prior anti-cancer treatment before baseline MRI; (2) non-primary breast cancer; (3) incomplete imaging or pathological data; (4) poor-quality MRI images; and (5) loss to follow-up immediately after surgery.

First, using survival data, we derived the optimal cutoffs for KA and δADC independently for both cohorts, and dichotomized patients into high and low groups accordingly. Subsequently, we compared the associations of KA and δADC with other clinicopathological variables. Next, we analyzed whether KA and δADC were independent prognostic factors. We also examined whether the prognostic effect of KA differed by molecular subtype using interaction tests. Survival differences between groups were also evaluated. Finally, we constructed two prediction models based on clinicopathological variables: Model 1 incorporated either KA or δADC, whereas Model 2 did not include these parameters. The ability of the models to stratify patients by risk was further validated.

### 2.2. MRI Acquisition and ADC Measurement

All patients underwent breast MRI before the initiation of NAC and after completion of NAC, prior to surgery. MRI was performed on a 3.0-T scanner (GE Medical Systems, LLC, Waukesha, WI, USA; Siemens Skyra, Erlangen, Germany) using a dedicated breast coil, with a slice thickness of 4 mm. The protocol included T1-weighted, T2-weighted, and diffusion-weighted imaging (DWI) sequences. DWI was acquired using a single-shot echo-planar imaging sequence with b-values of 0 and 800 s/mm^2^. ADC maps were automatically generated by the imaging workstation using a mono-exponential model. To measure ADC values, T2WI, DWI, ADC, and contrast-enhanced T1WI sequences were first imported into the workstation, and the solid enhancing component of the lesion was identified. Subsequently, a circular region of interest (ROI) measuring 50 mm^2^ was placed on the solid component at the largest cross-section of the lesion, avoiding necrotic, hemorrhagic, and cystic areas. The lowest ADC value within the ROI was recorded, which was evaluated for reliability via the intraclass correlation coefficient (ICC), and the NAC-induced ADC change (δADC) was calculated as post-ADC−pre-ADC. All ADC measurements were performed by two experienced radiologists blinded to clinical outcomes. In cases of disagreement, a third senior radiologist made the final decision [9].

### 2.3. Pathological Assessment

The following variables were collected: age, estrogen receptor status, progesterone receptor status, HER2 status, Ki67 index, P53, T-stage, N-stage, M-stage, and whether pCR was achieved.

Molecular markers were determined based on immunohistochemistry (IHC) and fluorescence in situ hybridization (FISH) results. ER and PR positivity were defined as ≥10% nuclear staining [10,11]. HER2 status was classified as follows: IHC 3+ or IHC 2+ with FISH positivity was considered HER2-positive; IHC 0 was considered HER2-negative; and all other cases were classified as HER2-low [12,13]. Molecular subtypes were defined as follows: tumors negative for ER, PR, and HER2 were classified as triple-negative breast cancer; tumors negative for ER and PR but positive for HER2 were classified as the HER2-overexpressing subtype; all remaining tumors were classified as the Luminal subtype. The KA was calculated as KA = [Ki67 (pre-treatment)]/[ADC (pre-treatment)].

All patients received 4 to 6 cycles of NAC followed by surgical resection. Surgical specimens were reviewed by specialized breast pathologists. pCR was defined as the absence of invasive carcinoma in both the breast and axillary lymph nodes, corresponding to ypT0/is ypN0 [3].

### 2.4. Follow-Up and Survival Outcomes

Patients were regularly followed up after treatment through outpatient visits or telephone interviews. Disease-free survival (DFS) was the survival outcome, which was defined as the time from surgery to recurrence, metastasis, death, or last follow-up. The follow-up cutoff date was 31 March 2024 for Cohort I and 21 May 2026 for Cohort II.

### 2.5. Development and Validation of Predictive Models

First, patients in Cohort I were randomly divided into a training set and a testing set at a ratio of 7:3, while patients in Cohort II served as a temporal validation set. Second, a survival prediction model was developed using Cox regression based on the training set. Variables with a *p*-value < 0.05 in the multivariate Cox regression analysis were selected as independent predictors. To ensure consistency, cutoff points for KA or δADC were derived in Cohort I and temporally validated in Cohort II. Third, model performance was evaluated using time-dependent receiver operating characteristic (ROC) curves with corresponding area under the curve (AUC) values, as well as the 95% confidence interval (CI) at relevant time points. Meanwhile, Kaplan–Meier (K-M) curves were used to evaluate the model’s risk stratification ability. Furthermore, to assess the contribution of KA or δADC to the model, we constructed models both with and without KA or δADC as independent variables (Model 1 and Model 2, respectively).

### 2.6. Statistical Analysis

All statistical analyses were performed using R software (version 4.5.3). The optimal cutoff values for KA and δADC were determined using the surv_cutpoint function. Continuous variables were compared using *t*-tests or Mann–Whitney U tests, and categorical variables were compared using chi-square or Fisher’s exact tests. Univariate and multivariate Cox proportional hazards regression models were used to evaluate prognostic factors for DFS. Variables with statistical significance in univariate analysis were entered into multivariate models. To predict survival risk, predictive models were developed using clinicopathological variables and incorporated with KA or δADC. Model performance was assessed by the time-dependent ROC curves with corresponding AUC values, as well as the 95% CI at relevant time points. Survival differences between groups were assessed using K-M curves, with comparisons performed using the log-rank test. A two-sided *p*-value < 0.05 was considered statistically significant.

## 3. Results

### 3.1. Patient Characteristics

**Figure 1:**
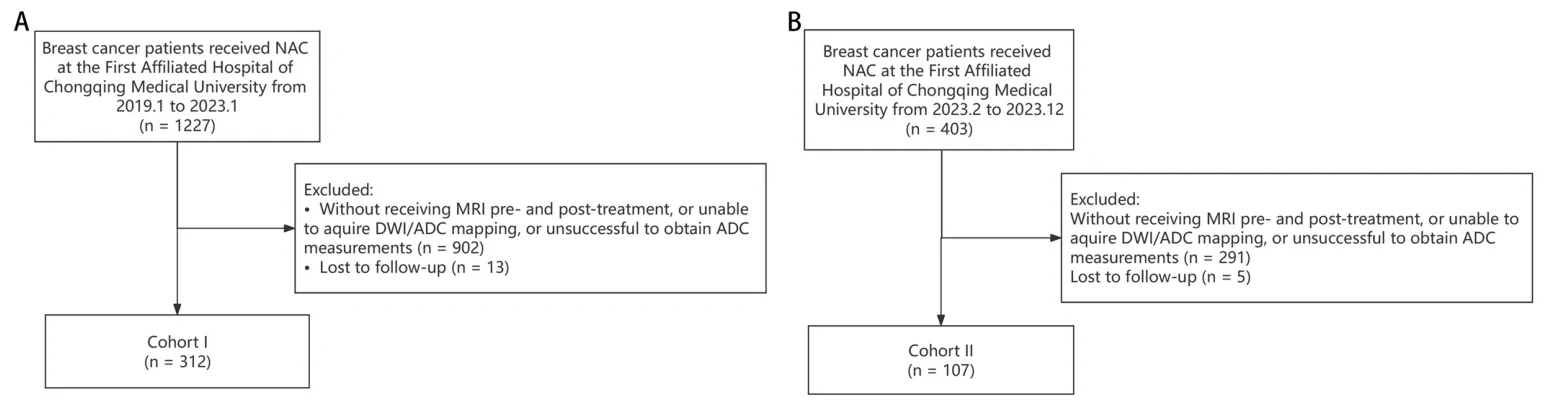
Patient screening flowchart for Cohort I (**A**) and Cohort II (**B**).

A total of 419 patients (Cohort I, *N* = 312; Cohort II, *N* = 107) were included. The patient screening process is shown in Figure 1. The ICC between two observers was 0.807 (95% CI: 0.771–0.838). The two cohorts were comparable in age, ER, PR, Ki67, T stage, N stage, pathological response, ADC, δADC, KA, and status (all *p* > 0.05). However, significant differences were observed for HER2 status, P53 expression, and M stage. Specifically, Cohort II had a higher proportion of HER2-low and M1 disease, but a lower proportion of P53 > 30%. Details were displayed in Table 1.

### 3.2. Association Between δADC, KA and Clinicopathological Variables

The surv_cutpoint function identified optimal cutoff values for δADC and KA as 0.582 and 57.471 in Cohort I, and −0.105 and 60.790 in Cohort II, respectively. Values above these thresholds were classified into the high group (*N* = 76), while those below were assigned to the low group (*N* = 236). Significant associations were found for ER status, PR status, HER2 status, and pathological response. Specifically, the high δADC group had a higher proportion of ER-negative, PR-negative, HER2-positive, and HER2-zero cases, but a lower proportion of HER2-low cases compared to the low δADC group. Notably, the high δADC group showed a significantly higher pCR rate than the low δADC group. The relative information was shown in Table 2.

**Figure 2:**
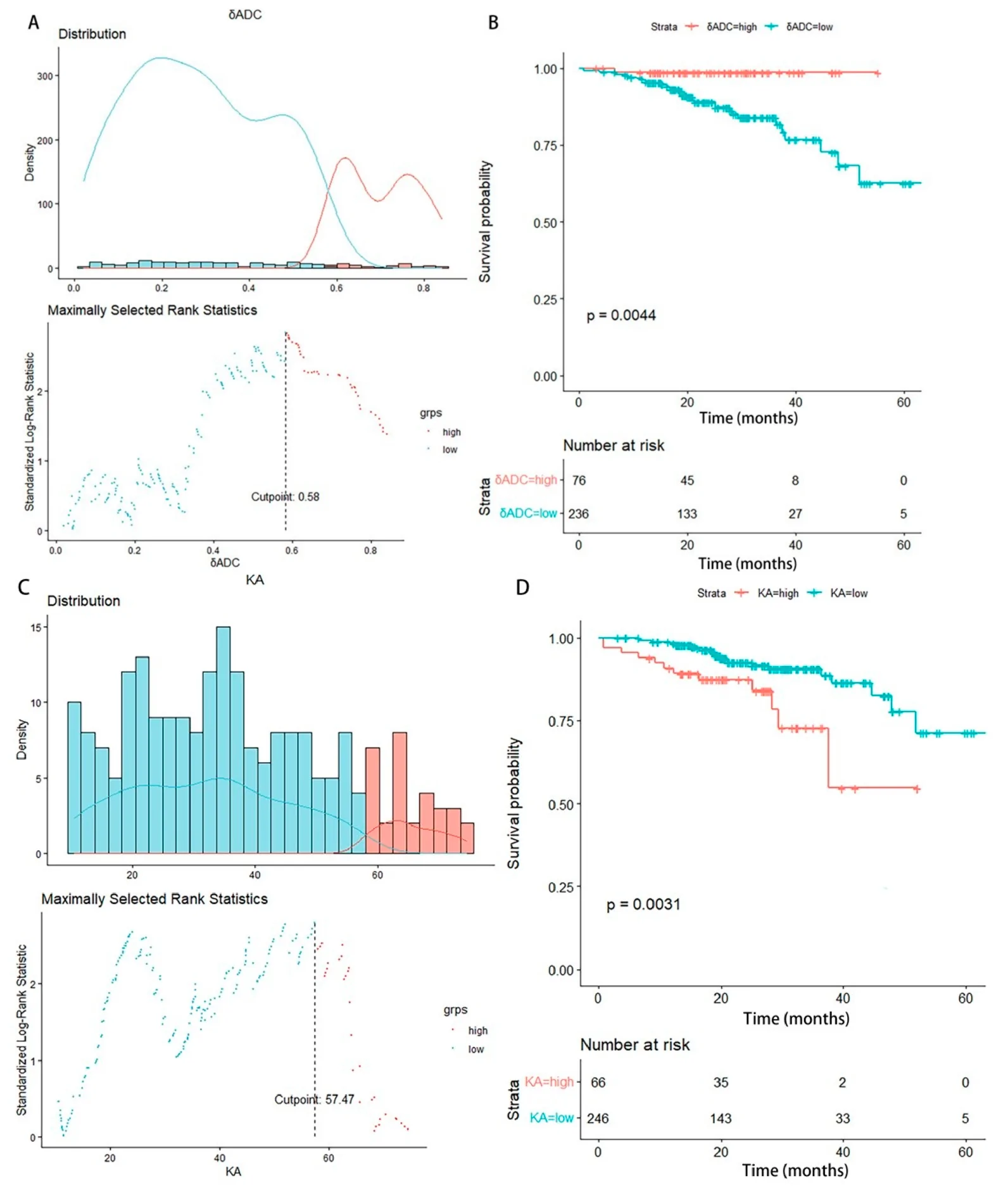

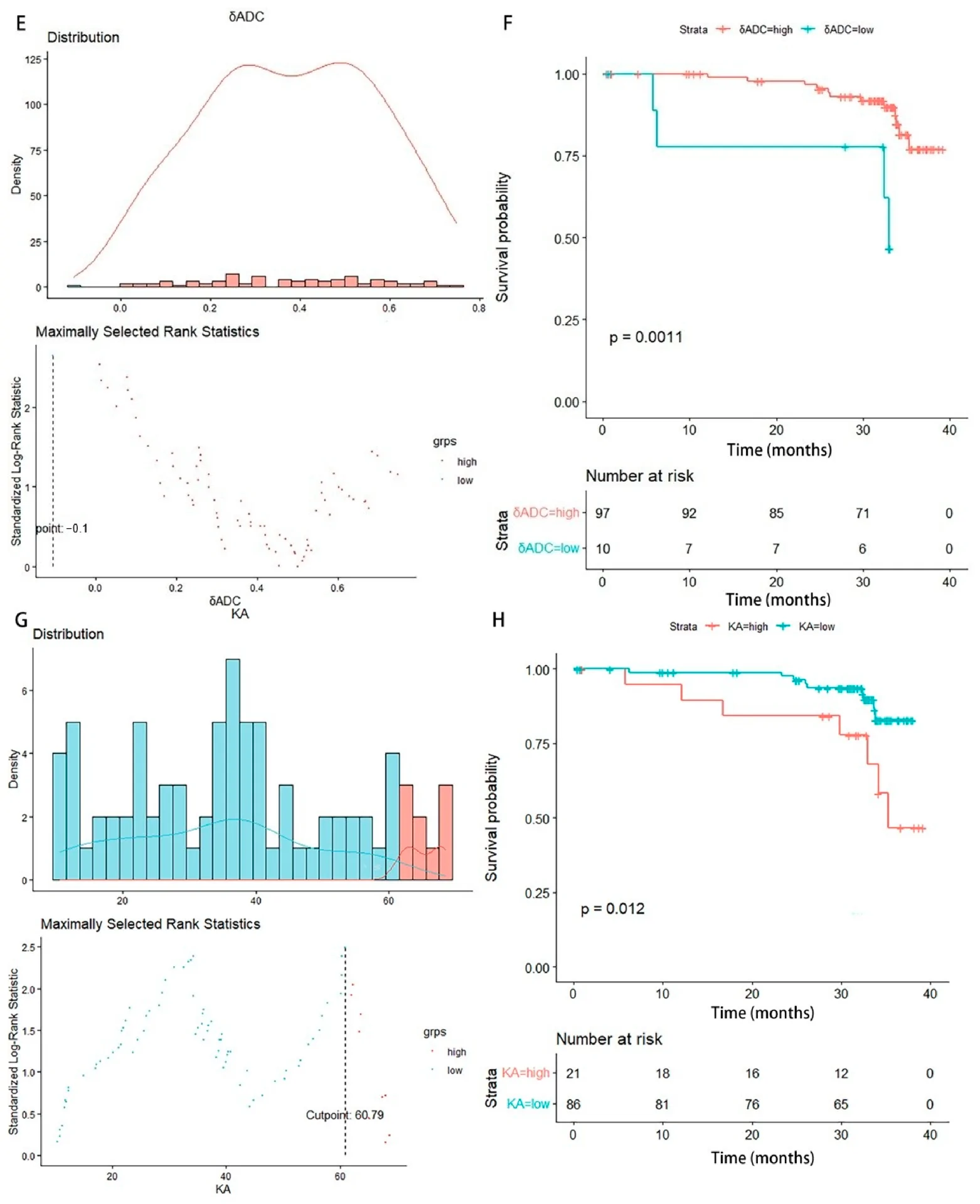
Cutoff selection and the subsequent survival differences between high and low groups for δADC (Cohort I: (**A**) and (**B**); Cohort II: (**E**) and (**F**), respectively). Cutoff selection and the subsequent survival differences between high and low groups for KA (Cohort I: (**C**) and (**D**); Cohort II: (**G**) and (**H**), respectively).

Furthermore, patients were stratified into high (*N* = 66) and low (*N* = 246) KA groups. Significant associations were found for HER2 status and Ki67. Specifically, the high KA group had a higher proportion of HER2-low and HER2-zero cases, but a lower proportion of HER2-positive cases compared to the low KA group. Notably, the high KA group was characterized by a marked predominance of Ki67 > 30%, whereas the low KA group had substantially higher proportions of Ki67 ≤ 20% and Ki67 20–30%. The relative information was shown in Table 3. Cutoff selection for δADC and KA and the subsequent survival differences between high and low groups were shown in Figure 2.

### 3.3. Prognostic Analysis

Univariable and multivariable Cox regression analyses were performed to identify factors associated with survival in Cohort I and Cohort II. In Cohort I, univariable analysis showed that low δADC and low KA were significant predictors. In multivariable analysis for Cohort I, low KA remained significant, while low δADC did not retain significance. In Cohort II, univariable analysis identified low δADC and low KA as significant predictors. In multivariable analysis for Cohort II, only low δADC remained significantly associated with survival. Detailed results were presented in Table 4. In Cox models including a KA × Subtype interaction term, the interaction *p* values were 0.932 in Cohort I and 0.994 in Cohort II, indicating that the prognostic effect of KA did not differ significantly across molecular subtypes.

**Figure 3:**
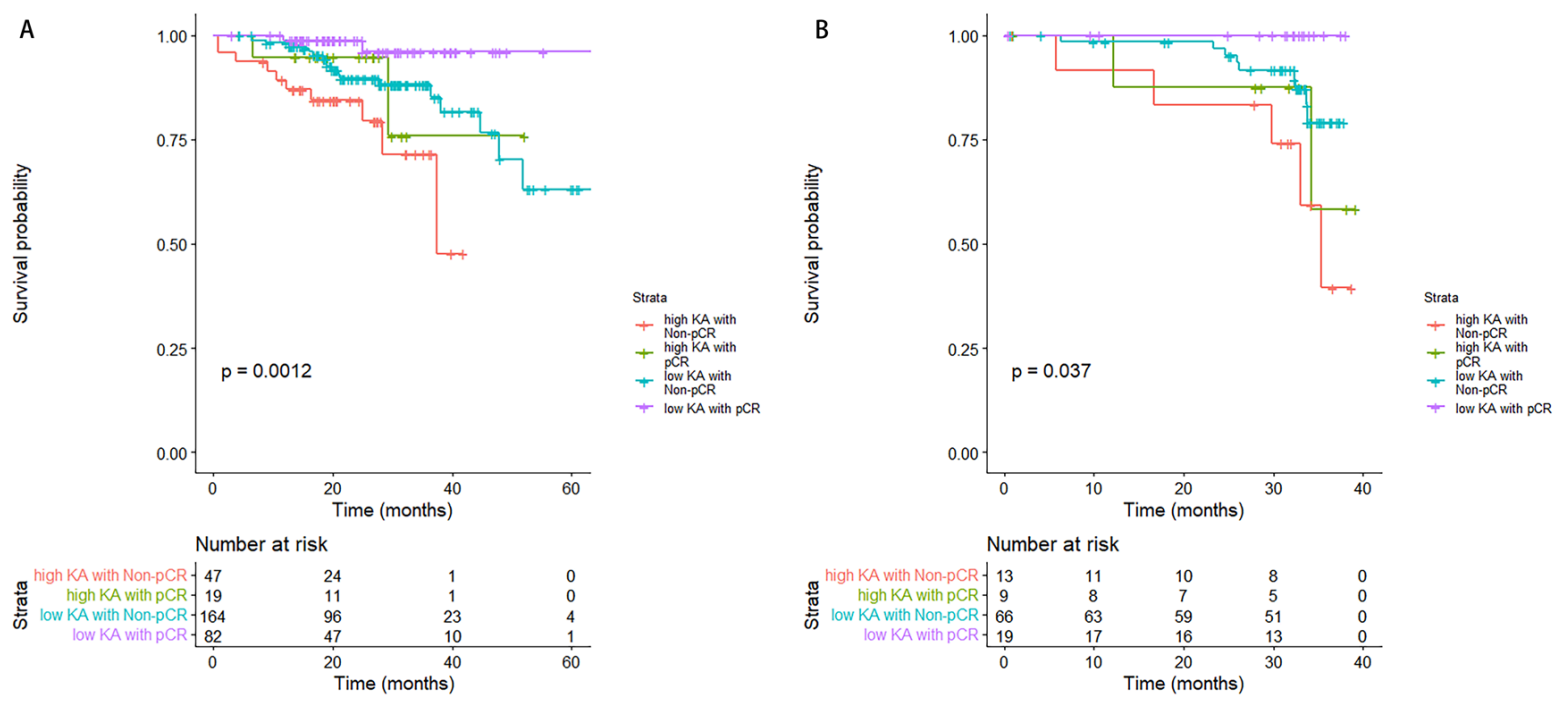
Survival differences between high and low KA groups according to pCR status (Panels (**A**) and (**B**) represent Cohort I and Cohort II, respectively.

Additionally, no significant survival difference was found between high and low KA groups among patients who achieved pCR. In contrast, among non-pCR patients, high KA was associated with significantly worse prognosis compared to low KA (*p* < 0.05 for both cohorts; Figure 3).

### 3.4. Cox Regression Model Performance

**Figure 4:**
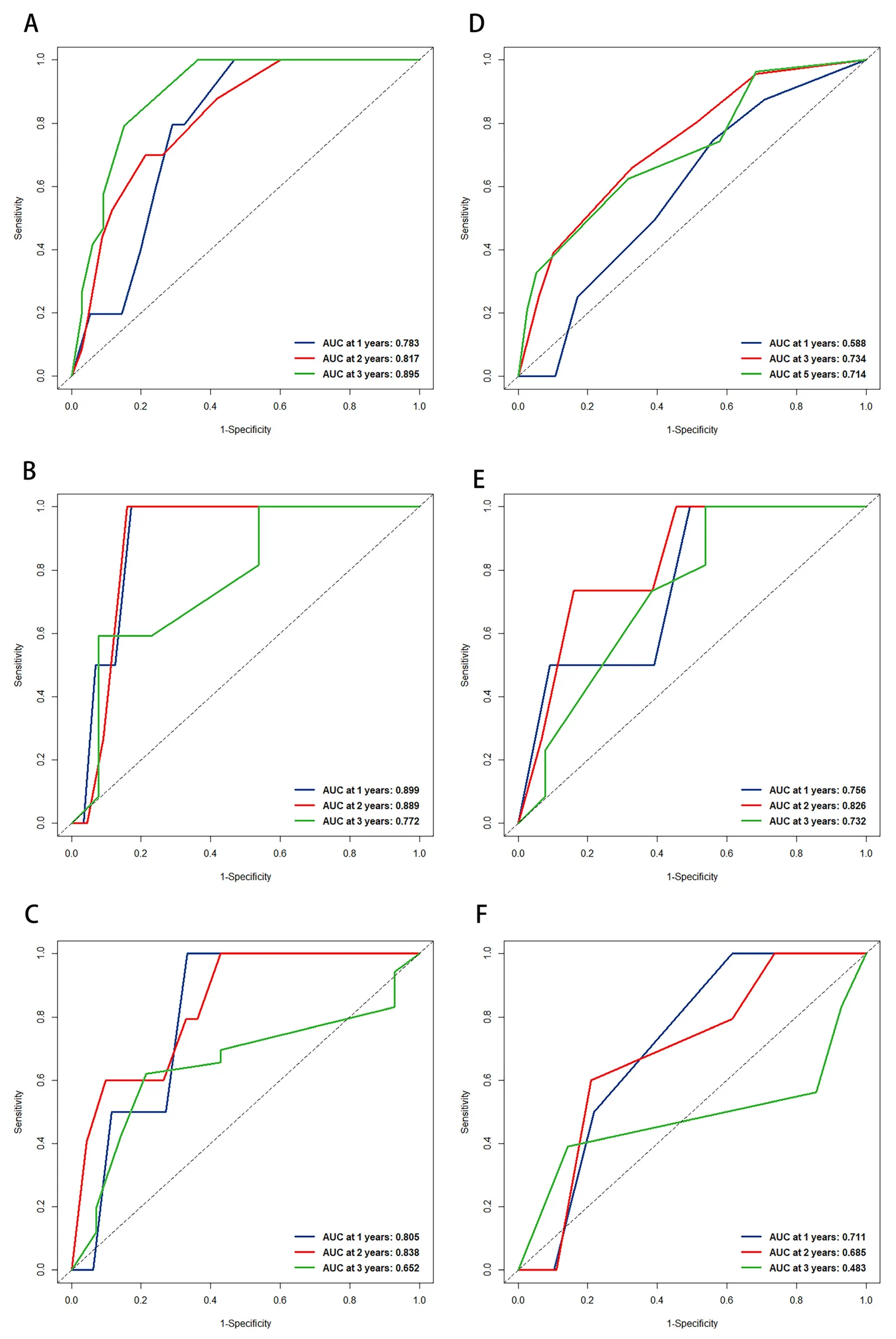
Time-dependent ROC curves of the model incorporating HER2, N-stage, and KA in the training set (**A**), testing set (**B**), and Cohort II (**C**), as well as those of the model incorporating only HER2 and N-stage in the training set (**D**), testing set (**E**), and Cohort II (**F**), at different time points.

**Figure 5:**
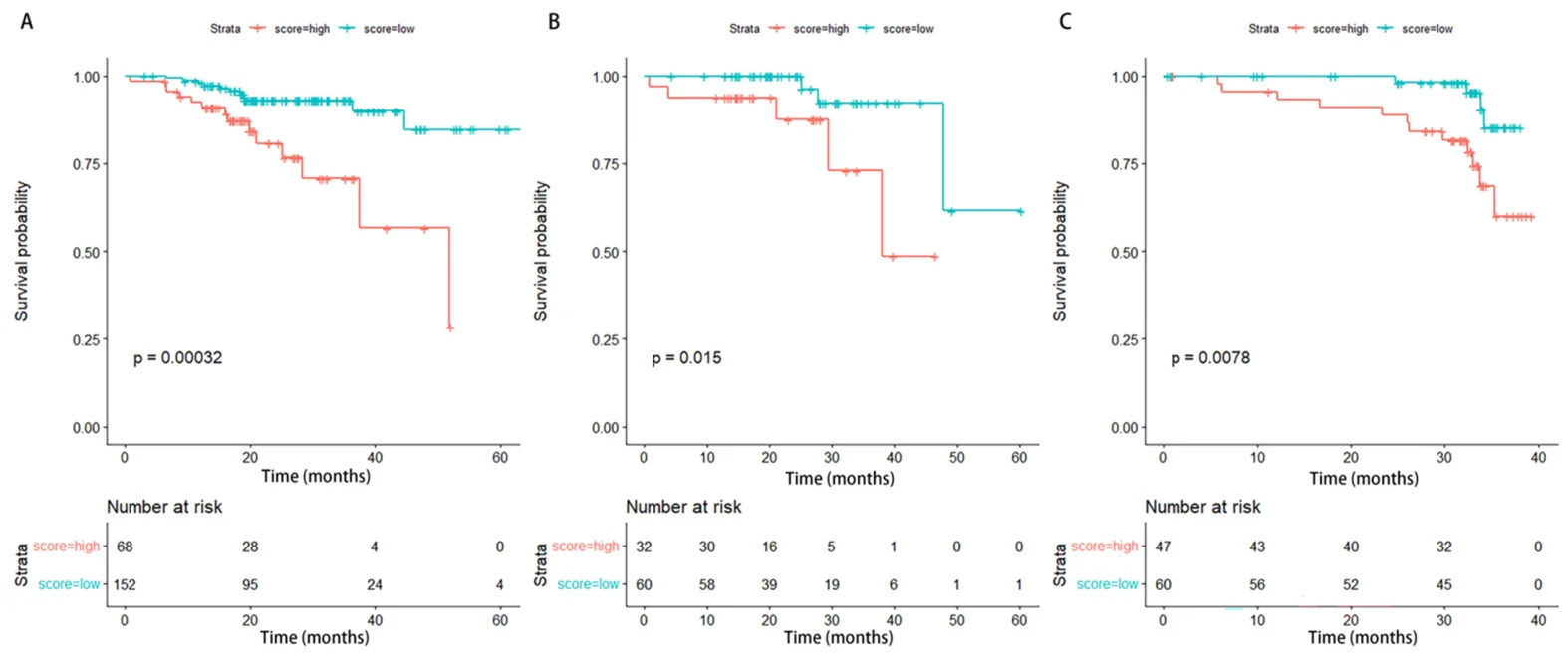
Based on the cutoff value determined by the model in the training set, patients were stratified into high-risk and low-risk groups. The survival differences between the two groups are shown for the training set (**A**), testing set (**B**), and Cohort II (**C**).

For Model 1, the proportional hazards assumption test showed that the *p* values for HER2, N, and KA were 0.872, 0.282, and 0.447, respectively. The C-index was 0.817 (95% CI: 0.752–0.882). The AUC values at 1, 2, and 3 years were 0.783 (95% CI: 0.640–0.925), 0.817 (95% CI: 0.691–0.944), and 0.895 (95% CI: 0.809–0.982) in the training set; 0.899 (95% CI: 0.820–0.968), 0.889 (95% CI: 0.813–0.965), and 0.772 (95% CI: 0.609–0.936) in the testing set; and 0.805 (95% CI: 0.643–0.966), 0.838 (95% CI: 0.692–0.984), and 0.652 (95% CI: 0.433–0.872) in Cohort II. For Model 2, the proportional hazards assumption test showed that the *p* values for HER2 and N were 0.807 and 0.409, respectively. The C-index was 0.702 (95% CI: 0.598–0.806). The AUC values at 1, 2, and 3 years were 0.588 (95% CI: 0.422–0.753), 0.734 (95% CI: 0.611–0.857), and 0.714 (95% CI: 0.588–0.840) in the training set; 0.756 (95% CI: 0.637–0.971), 0.826 (95% CI: 0.697–0.955), and 0.732 (95% CI: 0.508–0.956) in the testing set; and 0.711 (95% CI: 0.521–0.901), 0.685 (95% CI: 0.489–0.881), and 0.483 (95% CI: 0.249–0.717) in Cohort II (Figure 4). Schoenfeld residual plots for both models showed no systematic trends over time, supporting the proportional hazards assumption (Supplementary Figure S1). Furthermore, model performance was further evaluated using the Brier score; calibration curves and decision curve analysis (DCA) were presented in Supplementary Figures S2, S3 and S4, respectively. These results consistently indicated that the integrated model outperformed clinicopathological variables alone, suggesting that the combination of imaging and biological markers improves prognostic stratification. In the training set, the surv_cutpoint function identified a predicted value of 0.03 from Model 1 as the cutoff for distinguishing between high-risk and low-risk groups. After applying this cutoff to the testing set and Cohort II, patients in the high-risk group had significantly worse prognosis than those in the low-risk group, indicating that Model 1 effectively stratifies patients into high-risk and low-risk categories (Figure 5).

## 4. Discussion

In this retrospective study, we investigated the prognostic value of the combined biomarker KA, defined as the ratio of pre-treatment Ki67 to pre-treatment ADC, and the post-treatment change in ADC (δADC) in breast cancer patients receiving NAC. Our findings first confirmed the existence of cutoff values for KA and δADC in breast cancer patients, above or below which patients showed significant differences in clinicopathological variables and prognosis. And further suggested that these two parameters captured complementary aspects of tumor biology, reflecting proliferative activity and treatment-related microstructural alterations, respectively. Notably, KA emerged as a significant predictor of survival, and its addition to a Cox-based model yielded superior risk stratification compared with clinicopathological features used in isolation.

Despite its established role as a proliferation index, Ki67 alone offered only a limited window into tumor aggressiveness, particularly when examined in the absence of microenvironmental cues and architectural context [6,14,15]. In addition, ADC provided a noninvasive measure of water diffusion, which was inversely related to tissue cellularity and extracellular space restriction [16,17,18]. Low ADC values have been associated with hypercellular tumors, higher histological grade, and aggressive biological behavior [19,20]. Therefore, combining Ki67 and ADC into a single index might provide a more integrated representation of both proliferative potential and tissue architecture than either marker alone.

In our study, the association between ADC change and treatment response was biologically plausible. In principle, an increase in ADC after therapy might indicate reduced cellular density and be associated with pCR [21,22,23]. This has been widely accepted in previous research. However, our findings differed between cohorts, which might be related to sample size, follow-up duration, or heterogeneity in treatment regimens and tumor biology. Moreover, δADC alone might not be strongly linked to baseline clinicopathological variables or prognosis, indicating that it may primarily represent dynamic treatment response rather than inherent tumor characteristics [24].

Specifically, the KA integrates two complementary tumor characteristics—Ki67 reflects the proliferative fraction—while ADC inversely correlates with cellular density and restricted water diffusion. A high KA marks highly proliferative, dense tumors, implying greater aggressiveness than either factor alone, which aligns with its prognostic value: elevated KA predicted poorer outcomes, especially in non-pCR patients, indicating chemoresistance and higher recurrence risk, whereas low KA suggested a more favorable profile. Notably, among pCR achievers, survival did not differ by KA level, supporting that its prognostic impact is lost once invasive disease is eradicated.

Finally, the predictive Cox model incorporating KA showed improved predictive performance over clinicopathological variables alone. Traditional prognostic factors such as ER, PR, HER2 status, and T/N stage, while valuable, did not fully capture tumor heterogeneity [25]. Our findings further suggested that integrating quantitative imaging with biomarker data could enable more personalized prognostic stratification [26,27]. The model’s ability to stratify patients into high- and low-risk groups suggested potential clinical utility for identifying patients who might benefit from more intensive surveillance or additional adjuvant treatment strategies after surgery. In particular, patients with high KA value might represent a subgroup with persistent biological risk despite standard treatment.

To the best of our knowledge, although previous studies have analyzed the relationship between ADC and Ki67, very few have combined these two parameters [28]. Hence, our study also had several implications for precision oncology. First, it supported the concept that multimodal biomarkers outperformed single-parameter indicators when evaluating prognosis [29]. Second, it highlighted the feasibility of using routinely available clinical data, pathology, and MRI-derived metrics to develop earlier prognostic tools [30]. The KA could be used to identify high-risk patients before neoadjuvant chemotherapy. High KA patients might be considered for more intensive NAC regimens, closer monitoring during treatment, enrollment in clinical trials, or post-NAC adjuvant treatment escalation. Low KA patients might avoid unnecessary treatment intensification. Third, it suggested that pre-treatment combined biological-imaging markers might help identify patients with an inherently aggressive phenotype before surgery [31].

Several limitations should be acknowledged. First, this was a retrospective, single-center study, which might introduce selection bias and limit generalizability. Second, the sample size was relatively modest, especially in Cohort II, which included only 107 patients with 16 survival events, which limited statistical power, especially for subgroup analyses. Third, MRI acquisition and ADC measurement, although standardized, remained susceptible to interobserver variability and technical heterogeneity. Fourth, the study did not fully account for all potential confounders, such as detailed NAC regimens, dose intensity, molecular subtype stratification, or other emerging biomarkers. Fifth, external validation from independent institutions was still needed before clinical application. Finally, while KA and δADC appeared promising, the biological mechanisms underlying their prognostic value warranted further translational investigation.

Despite these limitations, our study provided evidence that the combination of Ki67 and ADC offered valuable prognostic information in breast cancer patients receiving NAC. These findings may contribute to more refined risk stratification and more personalized post-treatment management.

## 5. Conclusions

In conclusion, the pre-treatment KA predicted prognosis in breast cancer patients and showed stable prognostic value across cohorts. A Cox model incorporating KA improved survival risk stratification over clinicopathological factors alone. Clinically, high KA, especially in non-pCR patients, supported intensified adjuvant therapy, closer imaging surveillance, and earlier trial enrollment, whereas low KA might justify treatment de-escalation to reduce toxicity and costs. Since Ki67 and ADC are routinely available from biopsy and pre-treatment MRI, calculating KA requires no extra procedures and can be easily integrated into existing workflows to guide individualized post-NAC management. Larger prospective studies are warranted to further validate its utility.

## Figures and Tables

**Table 1:** Baseline characteristics of patients in the two cohorts.

		Cohort I (*N* = 312)	Cohort II (*N* = 107)	*p*
Age	Median (IQR)	50.5 (43.0 to 56.0)	52.0 (44.5 to 57.0)	0.181
ER	Negative	163 (52.2%)	54 (50.5%)	0.837
	Positive	149 (47.8%)	53 (49.5%)	
PR	Negative	214 (68.6%)	69 (64.5%)	0.508
	Positive	98 (31.4%)	38 (35.5%)	
HER2	HER2-low	101 (32.4%)	54 (50.5%)	<0.001
	HER2-positive	140 (44.9%)	28 (26.2%)	
	HER2-zero	71 (22.8%)	25 (23.4%)	
Ki67	≤20	97 (31.1%)	35 (32.7%)	0.701
	20~30	92 (29.5%)	27 (25.2%)	
	>30	123 (39.4%)	45 (42.1%)	
P53	>30	154 (49.4%)	35 (32.7%)	0.004
	≤30	158 (50.6%)	72 (67.3%)	
T	T1 + T2	228 (73.1%)	75 (70.1%)	0.638
	T3 + T4	84 (26.9%)	32 (29.9%)	
N	N0 + N1	204 (65.4%)	73 (68.2%)	0.677
	N2 + N3	108 (34.6%)	34 (31.8%)	
M	M0	303 (97.1%)	99 (92.5%)	0.048
	M1	9 (2.9%)	8 (7.5%)	
response	Non-pCR	211 (67.6%)	79 (73.8%)	0.281
	pCR	101 (32.4%)	28 (26.2%)	
ADC	Median (IQR)	0.9 (0.8 to 1.0)	0.9 (0.8 to 1.0)	0.460
δADC	Median (IQR)	0.3 (0.1 to 0.6)	0.4 (0.2 to 0.6)	0.573
KA	Median (IQR)	34.9 (21.1 to 53.7)	36.7 (21.7 to 56.6)	0.653
status	0	279 (89.4%)	91 (85%)	0.298
	1	33 (10.6%)	16 (15%)	
DFS	Median (IQR)	21.4 (16.4 to 31.6)	32.7 (29.2 to 34.1)	<0.001

**Table 2:** Association of δADC with other features in the two cohorts.

		Cohort I			Cohort II		
Name	Levels	High (*N* = 76)	Low (*N* = 236)	*p*	High (*N* = 97)	Low (*N* = 10)	*p*
Age	Median (IQR)/Mean ± SD	51.0 (45.0 to 57.0)	50.0 (42.0 to 56.0)	0.257	51.3 ± 10.2	52.2 ± 11.8	0.785
ER	Negative	49 (64.5%)	114 (48.3%)	0.020	48 (49.5%)	6 (60%)	0.742
	Positive	27 (35.5%)	122 (51.7%)		49 (50.5%)	4 (40%)	
PR	Negative	60 (78.9%)	154 (65.3%)	0.036	62 (63.9%)	7 (70%)	1.000
	Positive	16 (21.1%)	82 (34.7%)		35 (36.1%)	3 (30%)	
HER2	HER2-low	10 (13.2%)	91 (38.6%)	<0.001	51 (52.6%)	3 (30%)	0.151
	HER2-positive	44 (57.9%)	96 (40.7%)		26 (26.8%)	2 (20%)	
	HER2-zero	22 (28.9%)	49 (20.8%)		20 (20.6%)	5 (50%)	
Ki67	≤20	25 (32.9%)	72 (30.5%)	0.726	35 (36.1%)	0 (0%)	0.030
	20~30	24 (31.6%)	68 (28.8%)		24 (24.7%)	3 (30%)	
	>30	27 (35.5%)	96 (40.7%)		38 (39.2%)	7 (70%)	
P53	>30	39 (51.3%)	115 (48.7%)	0.795	29 (29.9%)	6 (60%)	0.076
	≤30	37 (48.7%)	121 (51.3%)		68 (70.1%)	4 (40%)	
T	T1 + T2	61 (80.3%)	167 (70.8%)	0.140	68 (70.1%)	7 (70%)	1.000
	T3 + T4	15 (19.7%)	69 (29.2%)		29 (29.9%)	3 (30%)	
N	N0 + N1	51 (67.1%)	153 (64.8%)	0.823	65 (67%)	8 (80%)	0.498
	N2 + N3	25 (32.9%)	83 (35.2%)		32 (33%)	2 (20%)	
M	M0	74 (97.4%)	229 (97%)	1.000	90 (92.8%)	9 (90%)	0.557
	M1	2 (2.6%)	7 (3%)		7 (7.2%)	1 (10%)	
KA	high	13 (17.1%)	53 (22.5%)	0.405	19 (19.6%)	3 (30%)	0.426
	low	63 (82.9%)	183 (77.5%)		78 (80.4%)	7 (70%)	
response	Non-pCR	20 (26.3%)	191 (80.9%)	<0.001	69 (71.1%)	10 (100%)	0.060
	pCR	56 (73.7%)	45 (19.1%)		28 (28.9%)	0 (0%)	

**Table 3:** Association of KA with other features in the two cohorts.

		Cohort I			Cohort II		
Name	Levels	High (*N* = 66)	Low (*N* = 246)	*p*	High (*N* = 22)	Low (*N* = 85)	*p*
Age	Median (IQR)/Mean ± SD	49.0 (42.0 to 57.0)	51.0 (43.0 to 56.0)	0.740	52.9 ± 10.9	51.0 ± 10.2	0.442
ER	Negative	42 (63.6%)	121 (49.2%)	0.051	14 (63.6%)	40 (47.1%)	0.251
	Positive	24 (36.4%)	125 (50.8%)		8 (36.4%)	45 (52.9%)	
PR	Negative	51 (77.3%)	163 (66.3%)	0.118	15 (68.2%)	54 (63.5%)	0.876
	Positive	15 (22.7%)	83 (33.7%)		7 (31.8%)	31 (36.5%)	
HER2	HER2-low	26 (39.4%)	75 (30.5%)	0.026	8 (36.4%)	46 (54.1%)	0.004
	HER2-positive	20 (30.3%)	120 (48.8%)		3 (13.6%)	25 (29.4%)	
	HER2-zero	20 (30.3%)	51 (20.7%)		11 (50%)	14 (16.5%)	
P53	>30	40 (60.6%)	114 (46.3%)	0.055	7 (31.8%)	28 (32.9%)	1.000
	≤30	26 (39.4%)	132 (53.7%)		15 (68.2%)	57 (67.1%)	
T	T1 + T2	53 (80.3%)	175 (71.1%)	0.182	15 (68.2%)	60 (70.6%)	1.000
	T3 + T4	13 (19.7%)	71 (28.9%)		7 (31.8%)	25 (29.4%)	
N	N0 + N1	43 (65.2%)	161 (65.4%)	1.000	13 (59.1%)	60 (70.6%)	0.438
	N2 + N3	23 (34.8%)	85 (34.6%)		9 (40.9%)	25 (29.4%)	
M	M0	64 (97%)	239 (97.2%)	1.000	21 (95.5%)	78 (91.8%)	1.000
	M1	2 (3%)	7 (2.8%)		1 (4.5%)	7 (8.2%)	
δADC	high	46 (69.7%)	161 (65.4%)	0.616	19 (86.4%)	78 (91.8%)	0.426
	low	20 (30.3%)	85 (34.6%)		3 (13.6%)	7 (8.2%)	
response	Non-pCR	47 (71.2%)	164 (66.7%)	0.580	13 (59.1%)	66 (77.6%)	0.136
	pCR	19 (28.8%)	82 (33.3%)		9 (40.9%)	19 (22.4%)	

**Table 4:** Analysis of prognostic factors for survival in the two cohorts.

		Cohort I		Cohort II	
		HR (Univariable)	HR (Multivariable)	HR (Univariable)	HR (Multivariable)
Age	Mean ± SD	1.04 (1.00–1.08, *p* = 0.059)		1.02 (0.97–1.07, *p* = 0.528)	
ER	Negative				
	Positive	0.89 (0.45–1.77, *p* = 0.744)		0.66 (0.25–1.79, *p* = 0.418)	
PR	Negative				
	Positive	0.53 (0.23–1.23, *p* = 0.142)		1.09 (0.39–2.99, *p* = 0.874)	
HER2	HER2-low				
	HER2-positive	0.31 (0.13–0.77, *p* = 0.011)	0.38 (0.15–0.94, *p* = 0.037)	0.83 (0.17–4.16, *p* = 0.824)	0.74 (0.15–3.74, *p* = 0.716)
	HER2-zero	1.07 (0.49–2.34, *p* = 0.867)	1.28 (0.58–2.82, *p* = 0.547)	3.79 (1.30–11.05, *p* = 0.015)	1.80 (0.50–6.43, *p* = 0.365)
Ki67	≤20				
	20~30	0.75 (0.27–2.11, *p* = 0.583)		2.65 (0.51–13.73, *p* = 0.244)	
	>30	2.00 (0.90–4.48, *p* = 0.091)		3.17 (0.68–14.73, *p* = 0.141)	
P53	>30				
	≤30	1.23 (0.62–2.46, *p* = 0.558)		0.72 (0.26–1.98, *p* = 0.523)	
T	T1 + T2				
	T3 + T4	1.57 (0.75–3.27, *p* = 0.228)		2.76 (1.03–7.38, *p* = 0.043)	2.20 (0.72–6.74, *p* = 0.168)
N	N0 + N1				
	N2 + N3	2.12 (1.05–4.28, *p* = 0.035)	2.13 (1.05–4.34, *p* = 0.037)	2.97 (1.10–8.01, *p* = 0.031)	2.38 (0.77–7.36, *p* = 0.133)
M	M0				
	M1	2.18 (0.51–9.20, *p* = 0.291)		1.99 (0.45–8.82, *p* = 0.366)	
δADC	high				
	low	0.35 (0.13–0.90, *p* = 0.030)	0.43 (0.16–1.14, *p* = 0.089)	5.85 (1.76–19.44, *p* = 0.004)	6.07 (1.75–21.06, *p* = 0.004)
KA	high				
	low	0.35 (0.17–0.72, *p* = 0.005)	0.40 (0.19–0.84, *p* = 0.015)	0.31 (0.12–0.85, *p* = 0.022)	0.48 (0.14–1.57, *p* = 0.223)
response	Non-pCR				
	pCR	0.28 (0.10–0.81, *p* = 0.018)	0.42 (0.14–1.24, *p* = 0.117)	0.44 (0.10–1.93, *p* = 0.276)	

## Data Availability

The data that support the findings of this study are not openly available due to reasons of sensitivity and are available from the corresponding author upon reasonable request.

## Supplementary Materials

The following supporting information can be downloaded at: https://www.mdpi.com/article/10.3390/curroncol33090534/s1. Figure S1. Schoenfeld residual plots for the model incorporating HER2, N-stage, and KA (A) and for the model incorporating only HER2 and N-stage (B); Figure S2: Brier scores of the two models at different time points in the training set (A), testing set (B), and Cohort II (C); Figure S3: Calibration curves for the Cox regression model including KA in the training set (A), testing set (B), and Cohort II (C). Calibration curves for the Cox regression model without KA in the training set (D), testing set (E), and Cohort II (F); Figure S4: DCA for the Cox regression model including KA in the training set (A), testing set (B), and Cohort II (C). DCA for the Cox regression model without KA in the training set (D), testing set (E), and Cohort II (F).

## References

[1] Bray F., Laversanne M., Sung H., Ferlay J., Siegel R.L., Soerjomataram I., Jemal A. (2024). Global cancer statistics 2022: GLOBOCAN estimates of incidence and mortality worldwide for 36 cancers in 185 countries. CA Cancer J. Clin..

[2] Giaquinto A.N., Sung H., Newman L.A., Freedman R.A., Smith R.A., Star J., Jemal A., Siegel R.L. (2024). Breast cancer statistics 2024. CA Cancer J. Clin..

[3] Korde L.A., Somerfield M.R., Carey L.A., Crews J.R., Denduluri N., Hwang E.S., Khan S.A., Loibl S., Morris E.A., Perez A. (2021). Neoadjuvant Chemotherapy, Endocrine Therapy, and Targeted Therapy for Breast Cancer: ASCO Guideline. J. Clin. Oncol..

[4] Curigliano G., Burstein H.J., Winer E.P., Gnant M., Dubsky P., Loibl S., Colleoni M., Regan M.M., Piccart-Gebhart M., Senn H.-J. (2017). De-escalating and escalating treatments for early-stage breast cancer: The St. Gallen International Expert Consensus Conference on the Primary Therapy of Early Breast Cancer 2017. Ann. Oncol..

[5] Cortazar P., Zhang L., Untch M., Mehta K., Costantino J.P., Wolmark N., Bonnefoi H., Cameron D., Gianni L., Valagussa P. (2014). Pathological complete response and long-term clinical benefit in breast cancer: The CTNeoBC pooled analysis. Lancet.

[6] Yerushalmi R., Woods R., Ravdin P.M., Hayes M.M., Gelmon K.A. (2010). Ki67 in breast cancer: Prognostic and predictive potential. Lancet Oncol..

[7] Pinker K., Moy L., Sutton E.J., Mann R.M., Weber M., Thakur S.B., Jochelson M.S., Bago-Horvath Z., Morris E.A., Baltzer P.A. (2018). Diffusion-Weighted Imaging with Apparent Diffusion Coefficient Mapping for Breast Cancer Detection as a Stand-Alone Parameter: Comparison with Dynamic Contrast-Enhanced and Multiparametric Magnetic Resonance Imaging. Investig. Radiol..

[8] Yao F.-F., Zhang Y. (2023). A review of quantitative diffusion-weighted MR imaging for breast cancer: Towards noninvasive biomarker. Clin. Imaging.

[9] Baltzer P., Mann R.M., Iima M., Sigmund E.E., Clauser P., Gilbert F.J., Martincich L., Partridge S.C., Patterson A., Pinker K. (2020). Diffusion-weighted imaging of the breast—A consensus and mission statement from the EUSOBI International Breast Diffusion-Weighted Imaging working group. Eur. Radiol..

[10] Allison K.H., Hammond M.E.H., Dowsett M., McKernin S.E., Carey L.A., Fitzgibbons P.L., Hayes D.F., Lakhani S.R., Chavez-MacGregor M., Perlmutter J. (2020). Estrogen and Progesterone Receptor Testing in Breast Cancer: ASCO/CAP Guideline Update. J. Clin. Oncol..

[11] Yu K., Cai Y., Wu S., Shui R., Shao Z. (2021). Estrogen receptor-low breast cancer: Biology chaos and treatment paradox. Cancer Commun..

[12] Wolff A.C., Hammond M.E.H., Allison K.H., Harvey B.E., Mangu P.B., Bartlett J.M.S., Bilous M., Ellis I.O., Fitzgibbons P., Hanna W. (2018). Human Epidermal Growth Factor Receptor 2 Testing in Breast Cancer: American Society of Clinical Oncology/College of American Pathologists Clinical Practice Guideline Focused Update. J. Clin. Oncol..

[13] Tarantino P., Hamilton E., Tolaney S.M., Cortes J., Morganti S., Ferraro E., Marra A., Viale G., Trapani D., Cardoso F. (2020). HER2-Low Breast Cancer: Pathological and Clinical Landscape. J. Clin. Oncol..

[14] Cheang M.C.U., Chia S.K., Voduc D., Gao D., Leung S., Snider J., Watson M., Davies S., Bernard P.S., Parker J.S. (2009). Ki67 index, HER2 status, and prognosis of patients with luminal B breast cancer. J. Natl. Cancer Inst..

[15] Näsiaho J., Nissi L., Ventelä S., Irjala H., Salmi M. (2026). Spatial single-cell analysis reveals tumor microenvironment signatures predictive of oral cavity cancer outcome. Cell Rep. Med..

[16] Hottat N.A., Badr D.A., Lecomte S., Besse-Hammer T., Jani J.C., Cannie M.M. (2023). Assessment of diffusion-weighted MRI in predicting response to neoadjuvant chemotherapy in breast cancer patients. Sci. Rep..

[17] Wang X., Ba R., Huang Y., Cao Y., Chen H., Xu H., Shen H., Liu D., Huang H., Yin T. (2024). Time-Dependent Diffusion MRI Helps Predict Molecular Subtypes and Treatment Response to Neoadjuvant Chemotherapy in Breast Cancer. Radiology.

[18] Hottat N.A., Badr D.A., Lecomte S., Besse-Hammer T., Jani J.C., Cannie M.M. (2022). Value of diffusion-weighted MRI in predicting early response to neoadjuvant chemotherapy of breast cancer: Comparison between ROI-ADC and whole-lesion-ADC measurements. Eur. Radiol..

[19] Bickel H., Clauser P., Pinker K., Helbich T., Biondic I., Brkljacic B., Dietzel M., Ivanac G., Krug B., Moschetta M. (2023). Introduction of a breast apparent diffusion coefficient category system (ADC-B) derived from a large multicenter MRI database. Eur. Radiol..

[20] Rahbar H., Partridge S.C., DeMartini W.B., Gutierrez R.L., Allison K.H., Peacock S., Lehman C.D. (2012). In Vivo Assessment of Ductal Carcinoma in Situ Grade: A Model Incorporating Dynamic Contrast-enhanced and Diffusion-weighted Breast MR Imaging Parameters. Radiology.

[21] Partridge S.C., Zhang Z., Newitt D.C., Gibbs J.E., Chenevert T.L., Rosen M.A., Bolan P.J., Marques H.S., Romanoff J., Cimino L. (2018). Diffusion-weighted MRI Findings Predict Pathologic Response in Neoadjuvant Treatment of Breast Cancer: The ACRIN 6698 Multicenter Trial. Radiology.

[22] Suo S., Yin Y., Geng X., Zhang D., Hua J., Cheng F., Chen J., Zhuang Z., Cao M., Xu J. (2021). Diffusion-weighted MRI for predicting pathologic response to neoadjuvant chemotherapy in breast cancer: Evaluation with mono-, bi-, and stretched-exponential models. J. Transl. Med..

[23] Pereira N.P., Curi C., Osório C.A.B.T., Marques E.F., Makdissi F.B., Pinker K., Bitencourt A.G.V. (2019). Diffusion-Weighted Magnetic Resonance Imaging of Patients with Breast Cancer Following Neoadjuvant Chemotherapy Provides Early Prediction of Pathological Response—A Prospective Study. Sci. Rep..

[24] Mohamed A.S.R., Abusaif A., He R., Wahid K.A., Salama V., Youssef S., McDonald B.A., Naser M., Ding Y., Joint Head and Neck Radiotherapy-MRI Development Cooperative (2023). Prospective validation of diffusion-weighted MRI as a biomarker of tumor response and oncologic outcomes in head and neck cancer: Results from an observational biomarker pre-qualification study. Radiother. Oncol..

[25] Zardavas D., Irrthum A., Swanton C., Piccart M. (2015). Clinical management of breast cancer heterogeneity. Nat. Rev. Clin. Oncol..

[26] Dialani V., Gaur S., Mehta T.S., Venkataraman S., Fein-Zachary V., Phillips J., Brook A., Slanetz P.J. (2016). Prediction of Low versus High Recurrence Scores in Estrogen Receptor–Positive, Lymph Node–Negative Invasive Breast Cancer on the Basis of Radiologic-Pathologic Features: Comparison with Oncotype DX Test Recurrence Scores. Radiology.

[27] Baxter G., Graves M., Gilbert F., Patterson A.J. (2019). A Meta-analysis of the Diagnostic Performance of Diffusion MRI for Breast Lesion Characterization. Radiology.

[28] Molinari C., Clauser P., Girometti R., Linda A., Cimino E., Puglisi F., Zuiani C., Bazzocchi M. (2015). MR mammography using diffusion-weighted imaging in evaluating breast cancer: A correlation with proliferation index. Radiol. Med..

[29] Goyal M., Marotti J.D., Workman A.A., Tooker G.M., Ramin S.K., Kuhn E.P., Chamberlin M.D., Diflorio-Alexander R.M., Hassanpour S. (2024). A multi-model approach integrating whole-slide imaging and clinicopathologic features to predict breast cancer recurrence risk. npj Breast Cancer.

[30] Chamming’s F., Ueno Y., Ferré R., Kao E., Jannot A.-S., Chong J., Omeroglu A., Mesurolle B., Reinhold C., Gallix B. (2018). Features from Computerized Texture Analysis of Breast Cancers at Pretreatment MR Imaging Are Associated with Response to Neoadjuvant Chemotherapy. Radiology.

[31] Jimenez J.E., Abdelhafez A., Mittendorf E.A., Elshafeey N., Yung J.P., Litton J.K., Adrada B.E., Candelaria R.P., White J., Thompson A.M. (2022). A model combining pretreatment MRI radiomic features and tumor-infiltrating lymphocytes to predict response to neoadjuvant systemic therapy in triple-negative breast cancer. Eur. J. Radiol..

