# Supplementary material for: Pretreatment Ki67-to-ADC Ratio Predicts Prognosis in Breast Cancer Patients Receiving Neoadjuvant Chemotherapy: A Retrospective Cohort Study
Source: Curr Oncol. 2026 Sep 2;33(9):534. doi: 10.3390/curroncol33090534 (PMC13605751; doi:10.3390/curroncol33090534)
Supplement: Supplementary file 1 [file curroncol-33-00534-s001.zip › curroncol-4375006-supplementary.pdf]

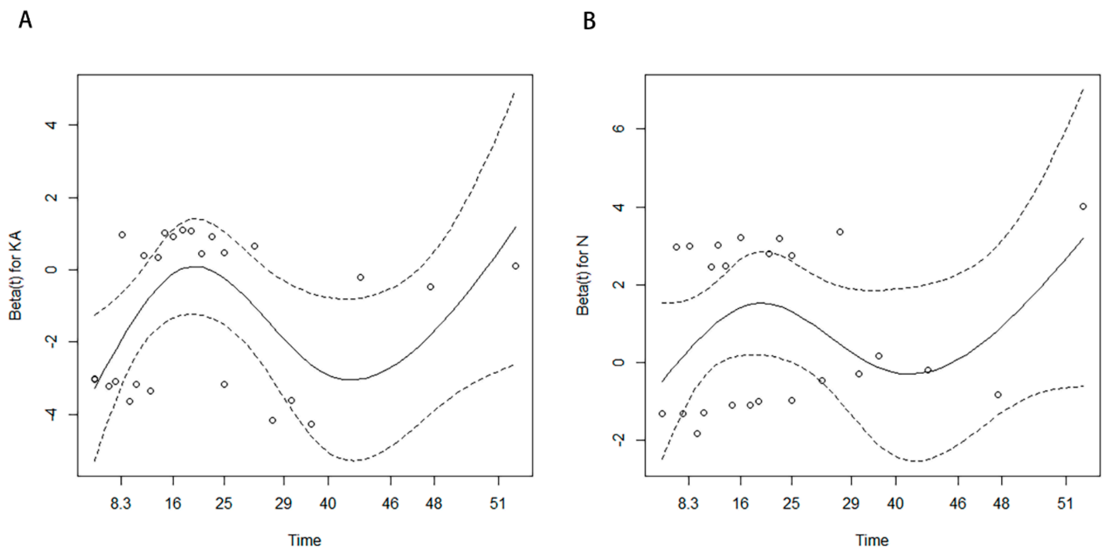

Supplementary Figure S1. Schoenfeld residual plots for the model incorporating HER2, N-stage, and KA (A) and for the model incorporating only HER2 and N-stage (B).

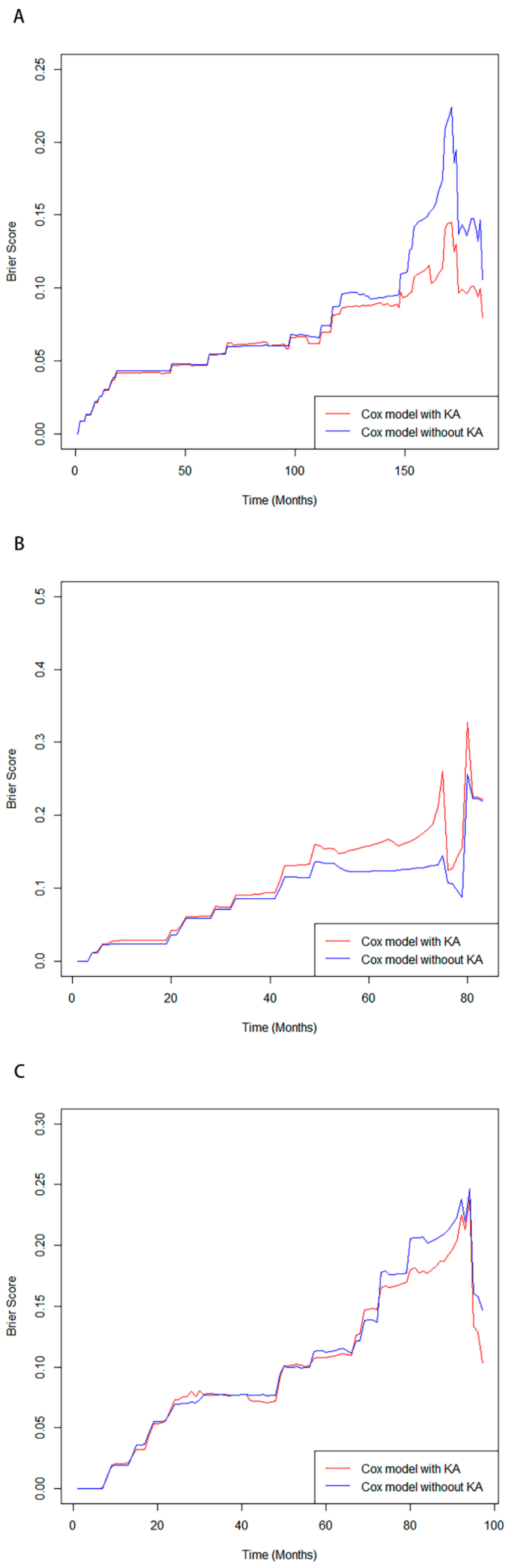

Supplementary Figure S2. Brier scores of the two models at different time points in the training set (A), testing set (B), and Cohort II (C).

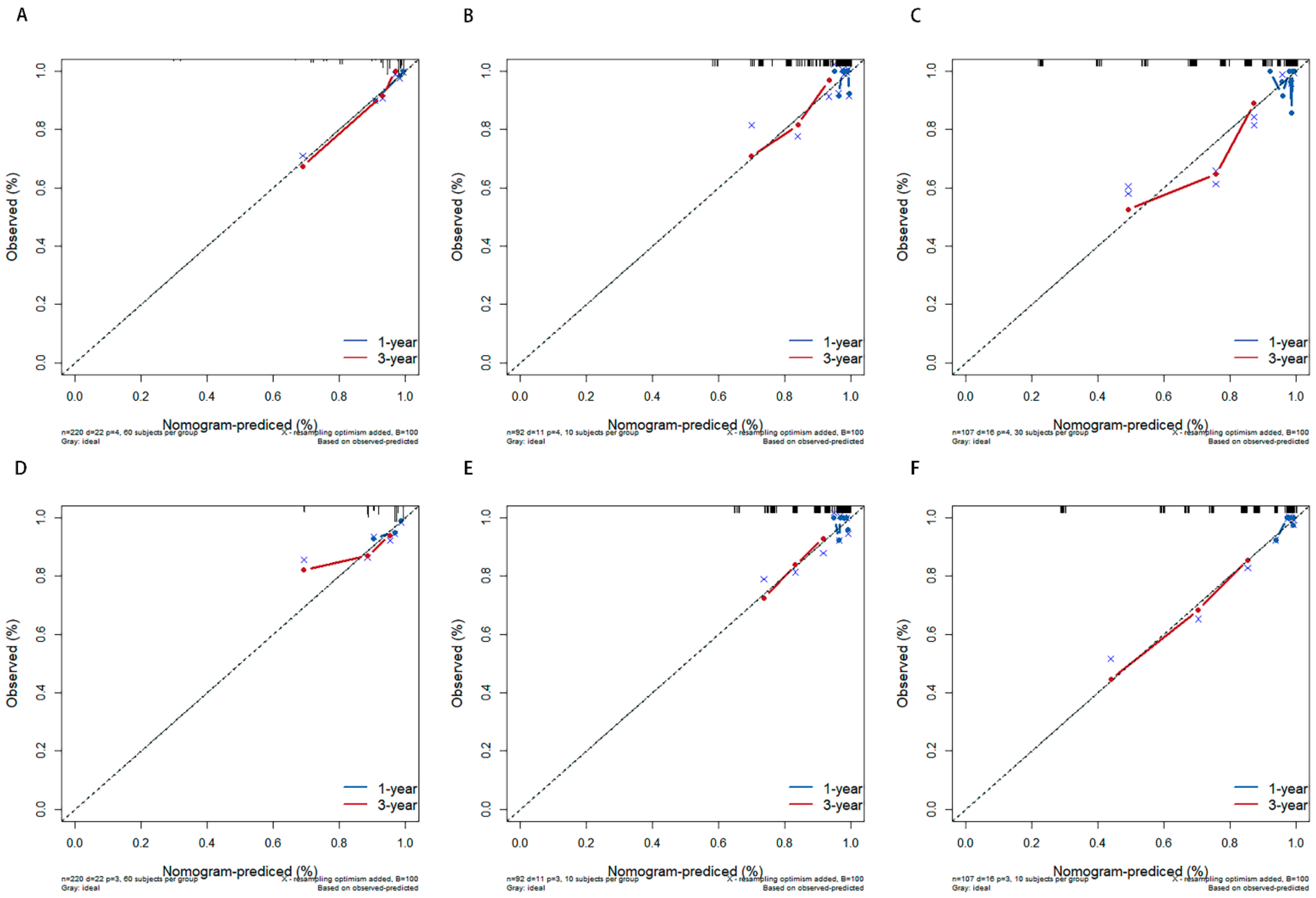

Supplementary Figure S3. Calibration curves for the Cox regression model including KA in the training set (A), testing set (B), and Cohort II (C). Calibration curves for the Cox regression model without KA in the training set (D), testing set (E), and Cohort II (F).

(Note: Calibration curves at 5 years are not shown because of an insufficient number of cases with 5-year follow-up.)

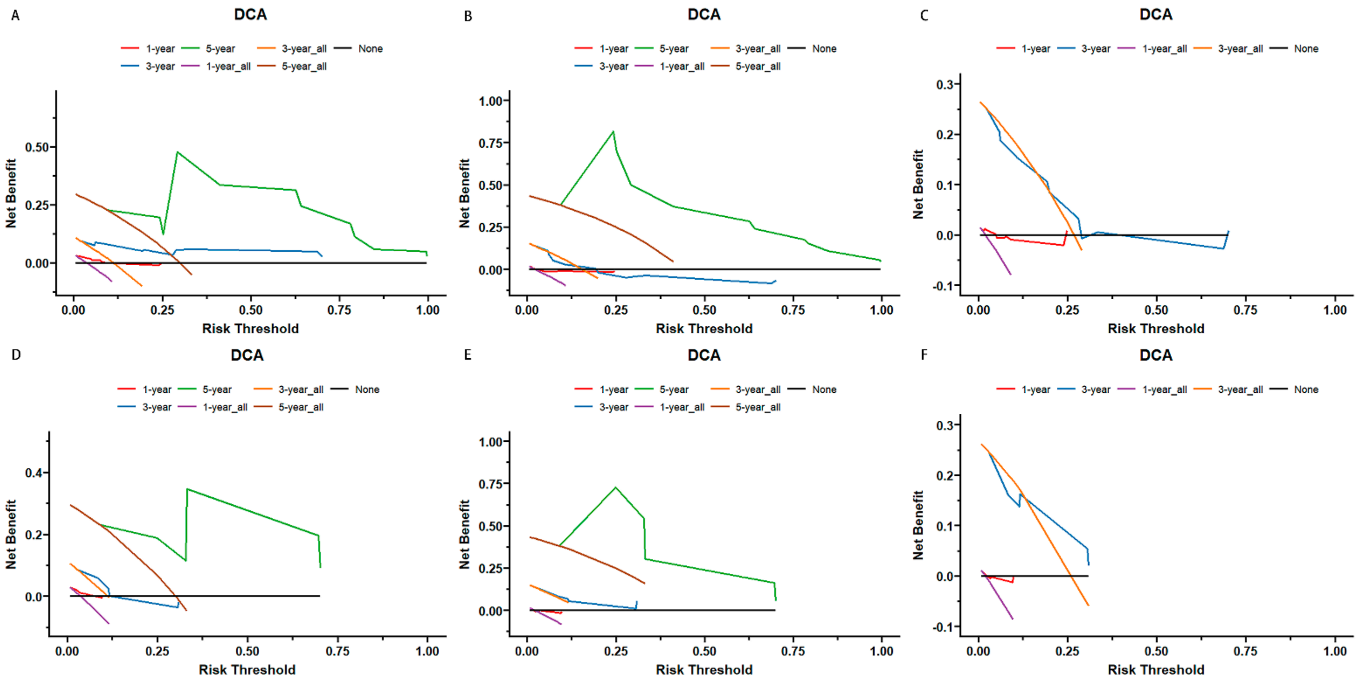

Supplementary Figure S4. DCA for the Cox regression model including KA in the training set (A), testing set (B), and Cohort II (C). DCA for the Cox regression model without KA in the training set (D), testing set (E), and Cohort II (F). (Note: Decision curves at 5 years are not shown in the Cohort II because of an insufficient number of cases with 5-year follow-up.)
